# Exploring the Antioxidant Effects and Periodic Regulation of Cancer Cells by Polyphenols Produced by the Fermentation of Grape Skin by *Lactobacillus plantarum* KFY02

**DOI:** 10.3390/biom9100575

**Published:** 2019-10-06

**Authors:** Jia Liu, Fang Tan, Xinhong Liu, Ruokun Yi, Xin Zhao

**Affiliations:** 1 Chongqing Collaborative Innovation Center for Functional Food, Chongqing University of Education, Chongqing 400067, China; liujia@cque.edu.cn (J.L.); liuxh@cque.edu.cn (X.L.); yirk@cque.edu.cn (R.Y.); 2 Chongqing Engineering Research Center of Functional Food, Chongqing University of Education, Chongqing 400067, China; 3 Chongqing Engineering Laboratory for Research and Development of Functional Food, Chongqing University of Education, Chongqing 400067, China; 4 Department of Public Health, Our Lady of Fatima University, Valenzuela 838, Philippines; 5 College of Biological and Chemical Engineering, Chongqing University of Education, Chongqing 400067, China

**Keywords:** *Lactobacillus plantarum* KFY02, polyphenols, HepG2, antioxidative, apoptotic

## Abstract

*Lactobacillus plantarum* KFY02 (LP-KFY02) was isolated from naturally fermented yoghurt in Xinjiang. We previously demonstrated that LP-KFY02 has good biological activity in vitro. In this study, LP-KFY02 was used to ferment grape skin, and the LP-KFY02 fermented grape skin extract solution (KFSE) was examined for its antioxidant ability in a human embryonic kidney (293T) cell oxidative damage model caused by H_2_O_2_ and its inhibitory effect on human hepatoma (HepG2) cells. The results showed that KFSE reduced the degree of oxidative damage in 293T cells, increased the relevant expression levels of superoxide dismutase (SOD), catalase (CAT), glutathione (GSH), and GSH-peroxidase (GSH-Px), and total antioxidant capacity (T-AOC), and decreased the expression levels of lactate dehydrogenase (LDH), malondialdehyde (MDA), and nitric oxide (NO). The expression of genes and proteins of SOD, CAT, GSH, and GSH-Px was up-regulated. In addition, KFSE-induced growth inhibition appeared to be through induction of cell-cycle arrest. This induction was accompanied by a reduction in the expression of cell-cycle genes, such as *cyclin-D1* and *CDK4*. In addition, KFSE induced gene expression of *p21*, the apoptosis gene wild-type *p53* and the caspase family. At the protein expression level, Bax and Caspase-8 were up-regulated, and the inflammatory marker Nuclear Factor Kappa-B (NF-κB) was down-regulated. The fermentation solution polyphenols were separated and identified as epicatechin gallate, coumarin, new chlorogenic acid, rutin, resveratrol, chlorogenic acid, rosmarinic acid, etc. by HPLC. Overall, these results demonstrate that KFSE significantly attenuated oxidative damage in 293T cells and inhibited tumor growth in HepG2 cancer cells, induces cell-cycle arrest and affects proteins involved in cell-cycle regulation and proliferation. This suggests that KFSE may also be explored as a neo-adjuvant to expansion of hepatoma.

## 1. Introduction

Epidemiological studies have shown that the incidence of hepatoma is increasing year by year due to environmental factors and lifestyle habits. The liver is an important organ of the human body. It mainly functions in detoxification. Many people are already in advanced stages when they are diagnosed with hepatoma. The symptoms of hepatoma are not obvious at an early stage. Patients can have no symptoms even after a long period of illness. When the disease develops to a certain extent, it will gradually produce pain in the liver area, loss of appetite, fatigue, weakness, and other symptoms. Ascites, hematemesis, coma, and other symptoms may occur in the advanced stage. The main treatment for hepatoma is surgical resection combined with comprehensive drug therapy or API-specific biological immunotherapy. The prevention of hepatoma should start with the cause. The preventive measures for hepatoma include abstaining from alcohol, reducing the pollution of pesticides, and supplementing trace elements such as selenium; selenium can induce differentiation and inhibit proliferation. In daily life, one can also eat certain liver-protecting foods, such as mushrooms and cabbages. In addition, clinical trials in patients with phenolic phytochemicals have shown that these drugs have potential chemo-preventative properties and low toxicity [1,2,3]. Since the symptoms of hepatoma are not significant, most patients are in the middle and late stages when they are diagnosed. Due to the abundant blood supply to the liver, cancer cells are easily transferred into and out of the organ, resulting in difficult treatment conditions. In a previous study, resveratrol was effective in inhibiting the growth of hepatoma [4]. Functional foods, such as kiwi, strawberry, and green tea, are rich in polyphenols and have been shown to be effective against cancer [5,6,7]. Polyphenols are known to have anti-proliferative and antioxidative effects on hepatoma cells.

Polyphenols are compounds found in plant foods that have potential health-promoting effects. These compounds are found in some common plant-based foods, such as tea, soy, red wine, vegetables, and fruits. Oxidative damage is an important cause of many chronic diseases, such as cardiovascular disease, cancer, and ageing. The antioxidant function of polyphenols can prevent these chronic diseases. In addition to the antioxidant effects of polyphenols, researchers have found that taking polyphenols while eating high-fat foods can reduce the health risks of high-fat foods. Grape contains components that are similar to those found in green tea, including polyphenol constituents such as resveratrol, gallic acid, catechin, quercetin, kaempferol, and several anthocyanins [8,9]. Studies have shown that polyphenols have antioxidant, anti-aging, and radiation-resistant properties [10]. For example, edible polyphenols enter the body, undergo a reduction reaction and are metabolized by the liver to the blood or other tissues to achieve a functional effect [11]. Our previous study also showed that the polyphenols in green tea inhibited hepatoma cell growth, induced apoptosis, and decreased the expression of genes that control apoptosis and the cell cycle in hepatoma (HepG2) cells.

Probiotics are a class of active microorganisms that can be beneficial to the host by altering the composition of the flora at a certain part of the host [12]. Probiotics and their metabolites have very high value, including promoting the digestion and absorption of nutrients, improving the body′s immunity, maintaining the balance of intestinal flora structure, improving meat quality, improving the body′s antioxidant level, inhibiting intestinal inflammation, and protecting intestinal mucosal barriers [13,14]. People with low immunity and weak bowel function have a good therapeutic response to probiotics. Furthermore, probiotics are also suitable for cancer patients who accepted chemotherapy or radiation therapy, because microbes in patients are easily killed by chemotherapy drugs, resulting in an imbalance of intestinal flora, so chemotherapy or radiotherapy should be actively supplemented with probiotics [15]. In patients with cirrhosis and periarthritis, probiotics can inhibit the production of fungi in the intestine, reduce endotoxin levels in the blood, reduce intestinal acidity, and prevent intestinal endotoxemia [16]. Our research used the *Lactobacillus plantarum* KFY02 (LP-KFY02, China General Microbiological Culture Collection Center, CGMCC No: 15638), which was isolated from the naturally fermented yoghurt in Korla, Xinjiang, by the Chongqing Collaborative Innovation Center for Functional Food. Early functional activity studies indicate that the strain have good resistance to gastric acid and bile salt activity. Moreover, the strain has a significant effect on the regulation of lipid metabolism in mice.

293T is a kind of cell line that is easy to transfect and construct model. Many oxidative stress model studies have selected 293T cells as sample models, and all had achieved good results [17]. Therefore, we selected 293T cells to establish an oxidative damage model caused by H_2_O_2_ and explore the effect of the solution fermented from grape skin by *Lactobacillus plantarum* KFY02 on the oxidative damage model. Moreover, HepG2 is a kind of liver cancer cell line with a short growth cycle and stable expression, which has made great contributions to the research of liver cancer diseases. In this study, we showed that the solution fermented from grape skin by *Lactobacillus plantarum* KFY02 can effectively alleviate the oxidative damage of 293T cells caused by H_2_O_2_ and significantly inhibits the growth and migration of HepG2 cells, primarily through the induction of cell-cycle arrest. These data, therefore, suggest that the solution fermented from grape skin by *Lactobacillus plantarum* KFY02 warrants further investigation as a treatment for therapy-resistant hepatoma.

## 2. Materials and Methods

### 2.1. Fermentation

Fresh grape skin was chilled in a vacuum and then pulverized into a powder. The grape skin powder was dissolved in sterile water at 1:10 (g:V) and inoculated with 1 × 10^7^ CFU/mL *Lactobacillus plantarum* KFY02 and the *Lactobacillus delbrueckii* subsp. *bulgaricus* (LB, China General Microbiological Culture Collection Center, CGMCC No. 1.16075) at 10% (V:V), respectively. Then, the mixture was fermented at 37 °C, at 100 rpm on a constant temperature shaker for 24, 48, 72, 96, and 120 h, and centrifuged at 12,000 rpm for 10 min. The supernatant was taken and stored at −80 °C (KFSE: The fermentation solution fermented by *Lactobacillus plantarum* (LP)-KFY02; BFSE: The fermentation solution fermented by *Lactobacillus delbrueckii* subsp. *bulgaricus*). In the control group, 10 g of grape skin powder was dissolved in 100 mL of 60% ethanol solution, and a water bath was used at 70 °C for 4 h. The precipitate was discarded and then evaporated to remove the ethanol and obtain grape skin extract (WS: The solution extracted by ethanol).

### 2.2. In Vitro Antioxidant Analysis

Previously described methods by Thaipong [18] and Roberta [19] were used to prepare 1,1-diphenyl-2-picrylhydrazyl (DPPH) radical-scavenging assay (Solarbio Life Sciences, Beijing, China) and 2,2′-azino-bis(3-ethylbenzothiazoline-6-sulfonic acid) (ABTS; Solarbio Life Sciences, Beijing, China) working fluid; the absorbance was measured using a multi-function micro-plate reader (Thermo Fisher Scientific, New York, NY, USA), and the ability of the fermentation solution to scavenge free radicals was calculated.

### 2.3. Culture of Cell

Human hepatoma (HepG2) cells and human embryonic kidney (293T) cells were purchased from the Cell Bank of the Chinese Academy of Sciences (Shanghai, China). The cell lines were cultured using DMEM supplemented with 10% fetal bovine serum (FBS; Sijiqing, Hangzhou Sijiqing Biological Engineering Materials Co., Ltd., Hangzhou, Zhejiang, China) 100 U/mL penicillin, and 100 μg/mL streptomycin (Gibco, Thermo Fisher Scientific) at 37 °C in a 5% CO_2_ atmosphere in a constant-temperature incubator (thermo371, Thermo Fisher Scientific, New York, NY, USA).

### 2.4. Oxidative Damage Model

293T cells at a density of 1 × 10^5^ were seeded in 96-well plates and incubated at 37 °C for 24 h. The original medium was removed, cells were stimulated with different concentrations of H_2_O_2_ (0, 100, 200, 300, 400, 500, 600, and 700 μmol/L), and after 1, 2, and 3 h, cell viability was measured by the CCK-8 (Solarbio Life Sciences, Beijing, China) method.

### 2.5. Apoptosis Model

HepG2 cells at a density of 1 × 10^5^ were inoculated into a 96-well culture plate and cultured in a cell culture incubator for 24 h. The original medium was removed, and different concentrations of KFSE were added to stimulate the cells for 48 h (0, 100, 200, 300, 400, 500, 600, and 700 μmol/L, we defined the fermented original solution to be 1 mol/L). Each condition was repeated three times. The cell viability was measured by the CCK-8 method.

### 2.6. Flow Cytometry

A total of 1 × 10^6^ 293T cells and HepG2 cells respectively plated in 6-well plates and starved for 24 h in serum-free medium before treatment, allowing cell division to synchronize. The cells in the oxidative damage group were treated with KFSE (200 μmol/L) for 48 h, and then H_2_O_2_ (100 μmol/L) was added to induce damage for 2 h. Apoptotic cells were treated with KFSE for 48 h. Then removed the medium and washed cells twice with pre-cooled phosphate-buffered saline (PBS). Flow cytometry (AccuriC6, BD Biosciences, San Jose, CA, USA) was used to detect apoptosis and the cell cycle according to the kit instructions (Yeasen, Shanghai, China).

### 2.7. RNA Extraction and qRT-PCR

293T and HepG2 cells were grown to 70–80% confluency for extraction and were treated with KFSE for 48 h. Then, H_2_O_2_ was used to induce oxidative damage for 2 h, and the apoptotic group was not treated. Cells were lysed using assay kits (BiaMaiKe; Beijing, China), and total RNA was extracted. RNA concentrations were determined using a micro-spectrophotometer (Nano-300, Hangzhou Allsheng Instruments Co., Ltd., Hangzhou, Zhejiang, China). One microgram of RNA was used for cDNA synthesis using the RevertAid First Strand cDNA Synthesis Kit (Thermo Fisher Scientific Baltics UAB, Vilnius, Lithuania). RT-PCR was performed in a HieffTM qPCR SYBR^®^ Green master mix (High Rox Plus; Yeasen, Shanghai, China) with a Step One Plus Real-Time PCR System (Thermo Fisher Scientific, New York, NY, USA). Test gene Ct values were normalized to Ct values of the housekeeping gene GAPDH, and fold differences compared to those of untreated controls were calculated. RT-qPCR was performed using the following cycling conditions: Predenaturation at 95 °C for 3 min, followed by 40 cycles of denaturation at 95 °C for 30 s, annealing at X °C for 30 s (X means the annealing temperature, which was determined by gradient PCR (A200 Gradient Thermal cycle, Zhejiang LongGene Scientific Instrument Co., Ltd., Hangzhou, Zhejiang, China)), and extension at 72 °C for 30 s. The corresponding annealing temperature information and primer sequence information are as shown in the Appendix A (Table A1).

### 2.8. Protein Isolation and Western Blotting Analysis

A total of 1 × 10^7^ 293T and HepG2 cells were cultured for 24 h, washed with cold PBS, and then lysed with 200 μL of radio immunoprecipitation assay (RIPA) buffer and 2 μL of phenylmethanesulfonyl fluoride (PMSF; Easy Bio, Beijing, China). Proteins (20 μg) were separated using 12% sodium dodecyl sulfate-polyacrylamide gel electrophoresis (SDS-PAGE; Whatman Schleicher and Schuell, Keene, NH, USA) and transferred to nitrocellulose membranes (PVDF, Thermo Fisher Scientific, Waltham, MA, USA). The membranes were blocked using 5% milk and probed with anti-SOD, CAT, GSH, GSH-Px, Bax, NF-κB, and Caspase8 (Invitrogen, Thermo Fisher Scientific, RockFord, USA; 1:1000 diluted in 5% milk) overnight at 4 °C. After washing with 1× TBST (Solarbio Life Sciences, Beijing, China) five times, the blots were treated with a second antibody (Cell Signaling Technology Inc., Danvers, MA, USA) for 1 h and washed with 1× TBST five times. Proteins were detected by enhanced chemiluminescence (ECL) HRP substrates (Solarbio Life Sciences, Beijing, China), and the expression was imaged using a Tanon 6200 Luminous Imaging Workstation (Tanon Science and Technology Co., Ltd., Shanghai, China). Western blot analysis was performed on total cell lysate. Semiquantitative analysis of protein expression was performed using ImageJ 1.44 software (National Institutes of Health, Bethesda, MD, USA).

### 2.9. High-Performance Liquid Chromatography (HPLC)

Standards for p-hydroxycinnamic acid, neochlorogenic acid, chlorogenic acid, rutin, polydatin, rosmarinic acid, and epicatechin gallate (EGCG; Shanghai Yuanye Biotechnology Co., Ltd. Shanghai, China) were weighed accurately, and a 0.1 mg/mL solution was prepared using methanol. Grape skin fermentation solution produced by lactic acid bacteria was extracted with a HyperSep C18 column (Thermo Scientific, 320 Rolling ridge Drive, Bellefonte, PA.), eluted with methanol–water (1:1, *V*/*V*), and filtered through a 0.22 μm filter. Fermentation components were detected (UltiMate3000 HPLC System, Thermo Fisher Scientific) using the following chromatographic conditions: Accucore C18 column (4.6 mm × 150 mm, 2.6 µm, Thermo Fisher Scientific); flow rate of 0.5 mL/min; detection wavelength of 285 nm; injection volume of 10 L; column temperature of 30 °C; collection time of 75 min; acetonitrile for mobile phase A (Thermo Fisher Scientific, USA); and 0.1% aqueous acetic acid solution for mobile phase B. The gradient elution conditions are shown in Table 1.

### 2.10. Statistical Analysis

The data were statistically analyzed by SPASS 17.0 and GraphPad Prism 7 statistical software. Experimental results are expressed as the mean ± standard deviation (SD). One-way ANOVA or *t*-test was used for comparison between groups. *p* ≤ 0.05 indicated that the difference was statistically significant.

## 3. Results

### 3.1. Antioxidant Activity In Vitro

**Figure 1:**
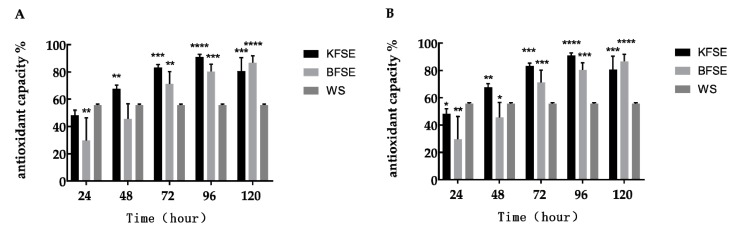
In vitro antioxidant activities of each experimental group. (**A**) Resistance of each experimental group to DPPH during different time intervals (24, 48, 72, 96, and 120 h). (**B**) Resistance of each experimental group to ABTS during different time intervals (24, 48, 72, 96, and 120 h). Values presented are the means ± standard deviation (*N* = 3/group). KFSE: The fermentation solution fermented by *Lactobacillus plantarum* (LP)-KFY02; BFSE: The fermentation solution fermented by *Lactobacillus delbrueckii* subsp. *bulgaricus*; and WS: The solution extracted by ethanol. * stands for *p* < 0.05, ** stands for 0.05 ≤ *p* < 0.01, **** stands for 0.01 ≤ *p* < 0.001, and **** stands for 0.001 ≤ *p* < 0.0001.

As depicted in Figure 1A,B, the antioxidant capacity of KFSE was stronger than that extracted by ethanol. KFSE achieved the maximum anti-DPPH and ABTS effects after 96 h of fermentation, reaching 90.84% and 81.38%, respectively. Moreover, the effect of KFSE was also stronger than that of BFSE and WS. This indicated that LP-KFY02 probiotics could significantly improve the antioxidant effect of the fermentation solution after fermenting the grape skin.

### 3.2. H_2_O_2_-Induced Oxidative Damage and Effects of KFSE on Human Embryonic Kidney (293T) Cell

**Figure 2:**
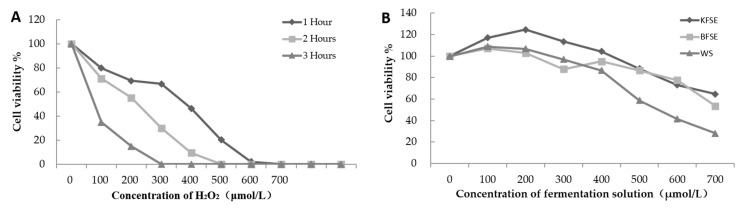
The oxidative damage model and the promotion of cell functions. (**A**) The trend in cell survival rate with time and concentration. (**B**) Effect of treatment with grape skin fermentation solution for 48 h on the growth viability of 293T cells. KFSE: The fermentation solution fermented by LP-KFY02; BFSE: The fermentation solution fermented by *Lactobacillus delbrueckii* subsp. *bulgaricus*; and WS: The solution extracted by ethanol.

As depicted in Figure 2A,B, H_2_O_2_ significantly inhibited the growth of 293T cells in time-dependent and concentration-dependent manners. The inhibitory effect of H_2_O_2_ at 200 μmol/L on 293T cells reached 54.67% after 2 h. In addition, in a concentration range (100–400 μmol/L), KFSE promoted the growth of 293T cells, and the proliferation rate of 293T cells with KFSE at a concentration of 200 μmol/L was 124.70%. This effect was better than that of BFSE and WS, and cell proliferation was inhibited when the concentration exceeded 400 μmol/L.

### 3.3. Effect of KFSE on Human Embryonic Kidney (293T) Cell Morphology

**Figure 3:**
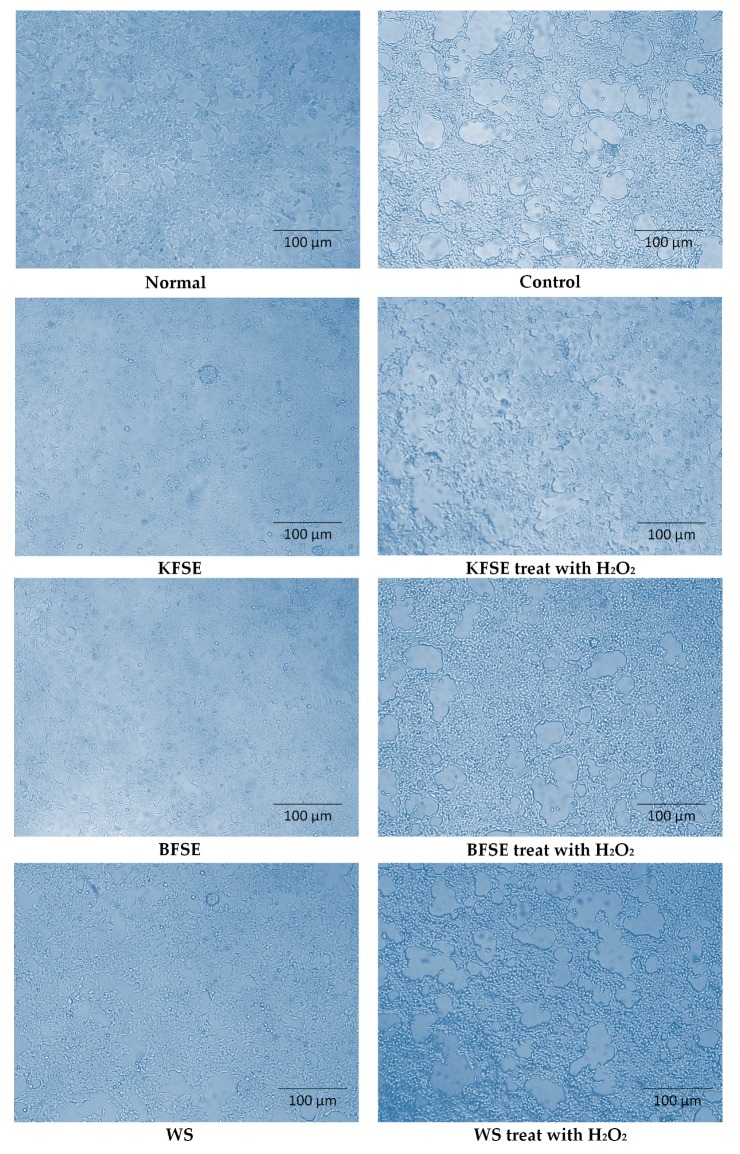
Effect of grape skin fermentation solution on 293T cell morphology (200×). KFSE: The fermentation solution fermented by LP-KFY02; BFSE: The fermentation solution fermented by *Lactobacillus delbrueckii* subsp. *bulgaricus*; and WS: The solution extracted by ethanol.

After culturing 293T cells with 200 μmol/L of KFSE for 48 h, the cells were exposed to H_2_O_2_ for 2 h at a concentration of 100 μmol/L, and the morphology of the cells was observed under an inverted microscope. As shown in Figure 3, the normal 293T cells were full of cells, rich in cytoplasm, numerous in number, and polygonal in shape, with pseudo-foot-like staggered around them. In the H_2_O_2_ induction model group, the cells were severely shrunken, the number of cells was reduced, and the damage was severe. The number of cells in the KFSE treatment group was increased, the cell state was good; in response to H_2_O_2_-induced oxidation, the protective effects of KFSE showed apparent improvement in cell body shrinkage, an increase in the number of cells, and a reduction in the degree of damage. BFSE and WS also had protective effects against oxidative damage but were not as effective as KFSE. The results indicated that KFSE could protect 293T cells from oxidative damage.

### 3.4. Oxidation Index of the Human Embryonic Kidney (293T) Cell

We demonstrated in Table 2 that compared with the oxidation index of the normal, in our damage model, the representative oxidation index levels of SOD, CAT, GSH, and GSH-Px were decreased and the levels of MAD, NO, and LDH were increased. Conversely, KFSE significantly increased the index levels of SOD, CAT, GSH, GSH-Px, and T-AOC and decreased the levels of MAD, NO, and LDH, which were damaged by H_2_O_2_. Moreover, there was no significant difference from the other groups. Therefore, we certified that KFSE could protect cells from oxidative damage and maintain the relevant indicators at normal levels.

### 3.5. KFSE Promotes SOD, CAT, GSH, and GSH-Px mRNA and Protein Expression in the Human Embryonic Kidney (293T) Cell

**Figure 4:**
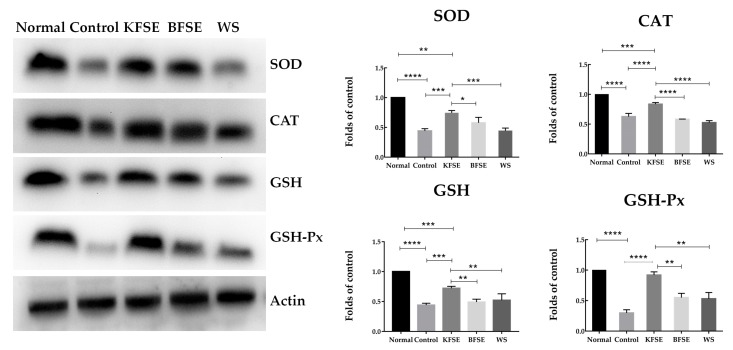
SOD, GSH, CAT, and GSH-Px protein expression in 293T cells. Values presented are the means ± standard deviation (*N* = 3/group). KFSE: The fermentation solution fermented by LP-KFY02; BFSE: The fermentation solution fermented by *Lactobacillus delbrueckii* subsp. *bulgaricus*; and WS: The solution extracted by ethanol. * stands for *p* < 0.05, ** stands for 0.05 ≤ *p* <0.01, **** stands for 0.01 ≤ *p* < 0.001, and **** stands for 0.001 ≤ *p* < 0.0001.

Table 3 shows that *SOD*, *CAT*, *GSH*, and *GSH-Px* mRNA (messenger RNA) levels decreased compared with normal levels. Compared with the control, KFSE significantly increased the expression of *SOD*, *CAT*, *GSH*, and *GSH-Px*, which play representative roles in oxidative damage of cells. Moreover, the expression of other products was also significantly affected after 48 h when compared with that of the control. The same result was also found in the protein expression level (Figure 4). We showed that KFSE increased SOD, CAT, GSH, and GSH-Px protein expression after H_2_O_2_ damage. In addition, BFSE and WS also significantly increased the protein levels of SOD, CAT, GSH, and GSH-Px compared with that of the model. Overall, our results confirmed the protective effect of KFSE in 293T oxidative damage.

### 3.6. KFSE Inhibits Human Hepatoma (HepG2) Cells Growth In Vitro

**Figure 5:**
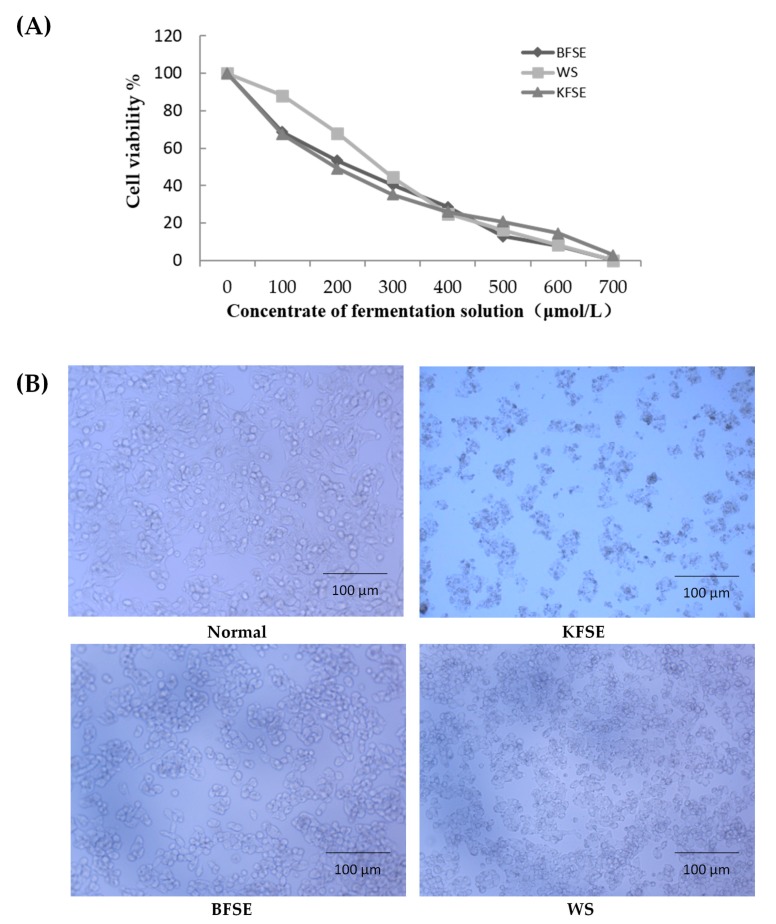
Effect of KFSE on HepG2 cell survival rate and the effect on cell morphology. (**A**) Effect of treatment with grape skin fermentation solution for 48 h on the survival rate of HepG2 cells. (**B**) Effect of treatment with grape skin fermentation solution for 48 h on the morphology of HepG2 cells (200×). KFSE: The fermentation solution fermented by LP-KFY02; BFSE: The fermentation solution fermented by *Lactobacillus delbrueckii* subsp. *bulgaricus*; and WS: The solution extracted by ethanol.

As depicted in Figure 5 KFSE significantly inhibited HepG2 cell growth by over 50% at a concentration of 300 μmol/L after 48 h and was sustained several concentrations. The significant inhibition was dose dependent (Figure 5A). The morphology of the cells is shown in Figure 5B. Compared with the morphology of the control group, the cells shrunk and shattered after KFSE treatment and reached over 50% apoptosis at 300 µmol/L for 48 h. The similar phenomena also appeared in the BFSC and WS. After a treatment with BFSE and WS for 48 h, we found that the morphology of cells was shrunk, the nucleus was obvious, the growth rate decreased, and cells exhibited varying degrees of shattered. We demonstrated significant growth inhibition in HepG2 liver cells.

### 3.7. KFSE Induces Cell Cycle Arrest in Human Hepatoma (HepG2) Cells

**Figure 6:**
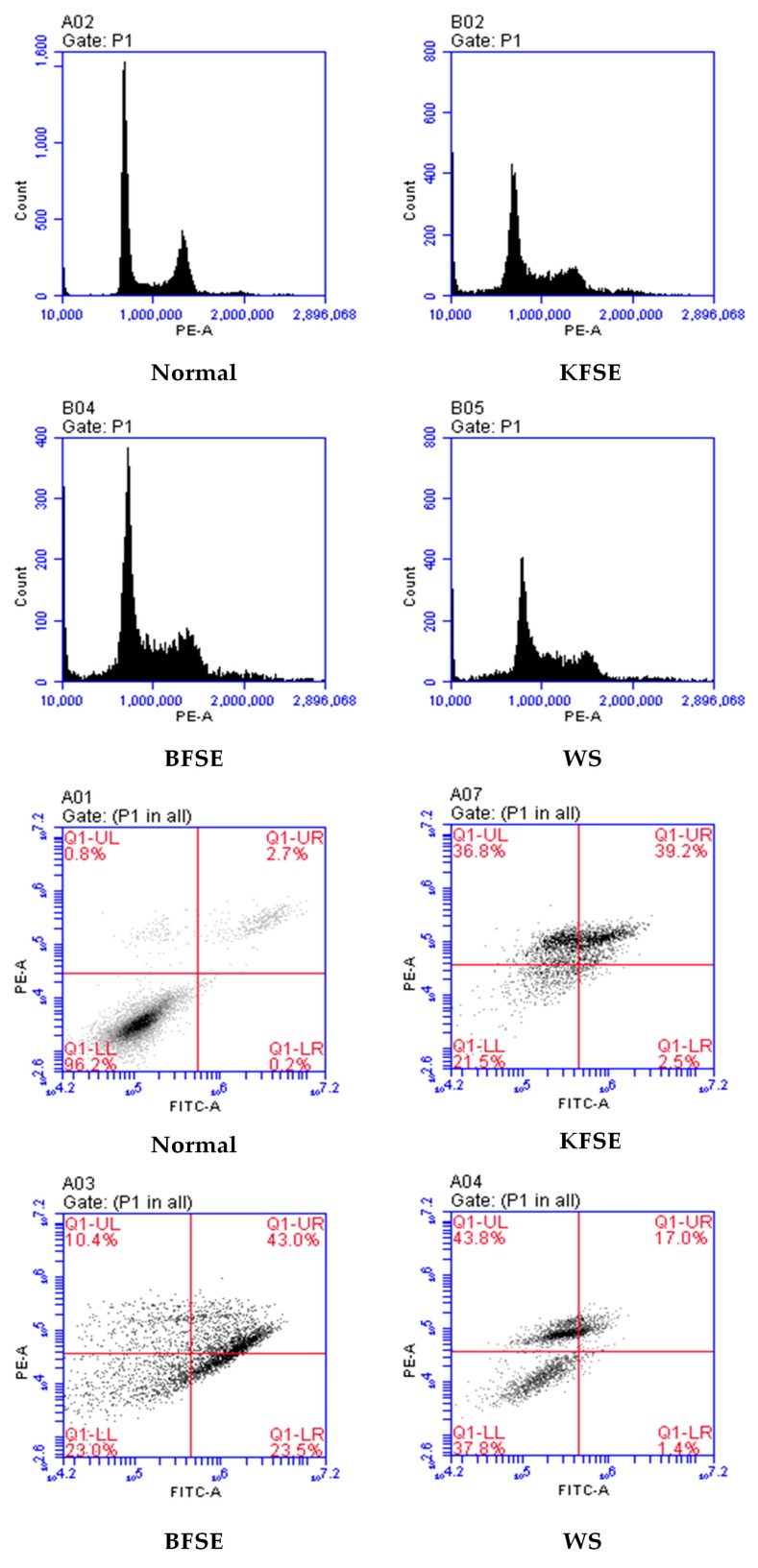
The effect of fermentation solution on the HepG2 cell cycle and apoptosis. KFSE: The fermentation solution fermented by LP-KFY02; BFSE: The fermentation solution fermented by *Lactobacillus delbrueckii* subsp. *bulgaricus*; and WS: The solution extracted by ethanol.

Cell cycle analysis demonstrated that KFSE significantly (*p* ≤ 0.05) arrested cells during the G1 to G2 phase transition at 300 μmol/L KFSE compared to that of the control after 48 h (Figure 6). Moreover, PI analysis demonstrated that KFSE also significantly induced apoptosis. This was confirmed by Annexin V-FITC/PI analysis (FITC: Fluorescein Isothiocyanate, PI: Propidium Iodide) (Figure 6). After KFSE treatment of HepG2 cells for 48 h, the detectable cell survival rate was only 24%, and more than 53.4% of cells were in advanced apoptosis and complete apoptosis.

### 3.8. Protein Expression and mRNA Expression in Human Hepatoma (HepG2) Cells

As shown in Table 4, Table 5 and Table 6 compared with levels in the normal group, the mRNA expression levels of *Bcl-2*, *cox-2*, *PCNA*, *cyclin-D1*, *C-myc*, *CDK4*, *NF-κB*, and *pRB1* in the KFSE group were significantly lower, and the expression levels of *Caspase-3*, *Caspase-7*, *Caspase-8*, *Caspase-9*, *p53*, *TNF-α*, and *p21* were higher. We showed that KFSE induced expression of *p53*, which is the key gene in apoptosis, and leads to the up-regulation of *p21* expression. The results were consistent with KFSE, which we found to promote apoptosis and blockade of hepatoma cells.

**Figure 7:**
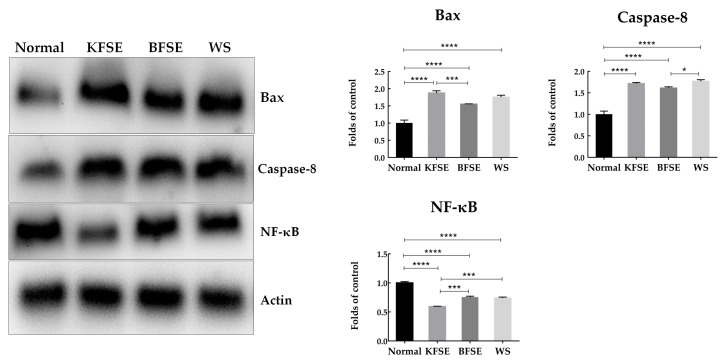
Bax, Caspase8, and NF-κB protein expression in HepG2 cells. Values presented are the means ± standard deviation (*N* = 3/group). KFSE: The fermentation solution fermented by LP-KFY02; BFSE: The fermentation solution fermented by *Lactobacillus delbrueckii* subsp. *bulgaricus*; WS: The solution extracted by ethanol. * stands for *p* < 0.05, ** stands for 0.05 ≤ *p* < 0.01, **** stands for 0.01 ≤ *p* < 0.001, and **** stands for 0.001 ≤ *p* < 0.0001.

The levels of Bax, Caspase-8, and NF-κB proteins determined by western blot analysis are shown in Figure 7. Compared with the levels of the normal group, the protein levels of Bax and Caspase-8 in the KFSE treatment group were significantly increased, and the level of NF-κB protein was decreased. The same results were also found in the BFSE and WS treatment groups, which significantly increased levels of the apoptotic proteins Bax and Caspase8 and decreased the protein expression of the inflammatory factor NF-κB.

### 3.9. Constituents of Fermentation Solution

**Figure 8:**
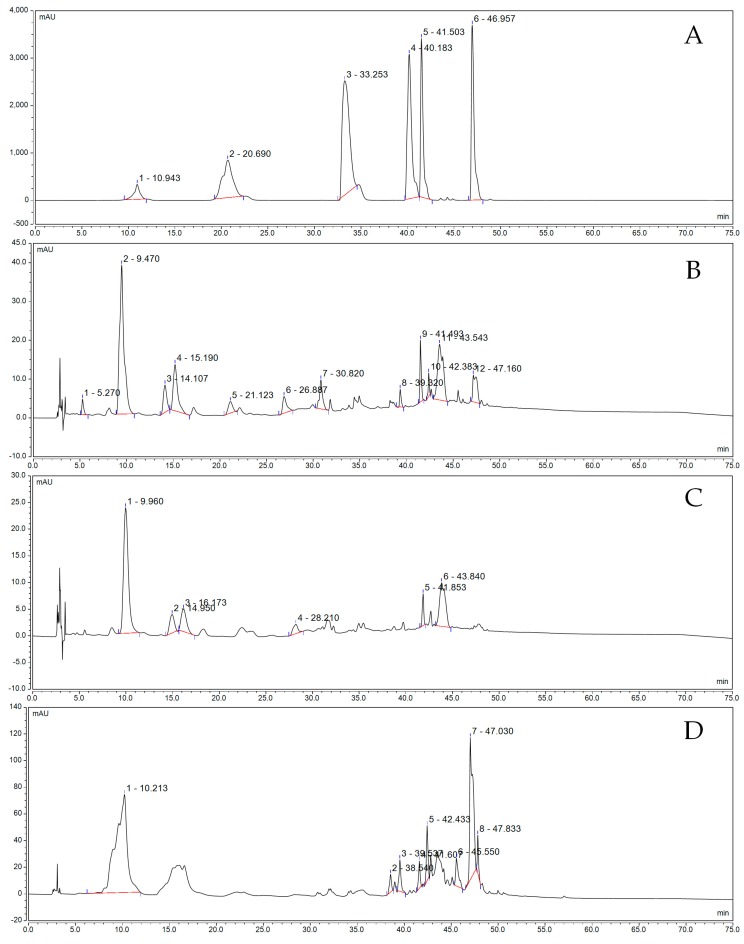
Polyphenol constituents of the fermented grape solution. (**A**) Standard chromatograms; (**B**) KFSE chromatograms; (**C**) BFSE chromatograms; and (**D**) WS chromatograms. 1: Epicatechin gallate (EGCG); 2: Coumarin; 3: New chlorogenic acid; 4: Rutin; 5: Resveratrol; and 6: Chlorogenic acid. KFSE: The fermentation solution fermented by LP-KFY02; BFSE: The fermentation solution fermented by *Lactobacillus delbrueckii* subsp. *bulgaricus*; and WS: The solution extracted by ethanol.

Figure 8 shows the polyphenol constituents of the fermented grape solution. As shown in Figure 8B, the total peak area of the fermentation solution fermented by LP-KFY02 was lower than that of the grape skin extract obtained from ethanol. Among them, the content of EGCG, rutin, and resveratrol in KFSE was the highest, reaching 25.54, 10.70, and 19.40 mAV*min, respectively (Figure 8B), the extract obtained from ethanol obtained the highest rutin content, reached 37.73 mAV*min (Figure 8D). However, the effective peak of KFSE fermentation solution was 94, the effective peak of ethanol extract was 83, and the active ingredient was more than ethanol extraction, while the fermentation result of BFSE only accumulated 59 effective peaks (Figure 8C). By standard reference comparison, the fermentation solutions included ECG, coumarin, new chlorogenic acid, rutin, resveratrol, chlorogenic acid, and rosmarinic acid (Figure 8A).

## 4. Discussion

This study examined whether KFSE was an antioxidant for human renal epithelial cells and an effective inhibitor of hepatoma in vitro. This study demonstrated that KFSE significantly inhibited cell growth of human hepatoma (HepG2) cells in vitro. This growth inhibition was accompanied by the induction of cell cycle arrest. Other studies have demonstrated significant growth-inhibitory effects by naturally occurring compounds in breast cancer cell lines. For example, Gupta et al. showed that Epigallocatechin gallate (EGCG) induced G1 phase arrest in human prostate carcinoma cells [20]. Comparable results were also observed after treatment with resveratrol in HT-29 cells [21] and quercetin in human osteosarcoma cells [22].

Oxidative damage is a key factor in the ageing, injury, and inflammation. Chronic diseases caused by oxidative damage, such as Alzheimer′s disease, inflammation, and cancer, have always threatened people′s lives. Bioflavonoids, polyphenols, vitamin C, anthocyanin, etc. are recognized as natural antioxidant products. It can effectively inhibit or alleviate the damage caused by oxidation and is of great significance for the treatment of various chronic diseases. Polyphenols are a group of compounds with a plurality of phenols per molecular unit and polyphenols are compounds that can be found in plants and have many health benefits. Many plants contain this type of compound and are most commonly found in flavonoids, phenolic acids, catechins, anthocyanins, isoflavones, quercetin, and resveratrol. Many studies have confirmed that these compounds always have a positive impact on human health.

Grapes and grape products, such as wine, grape seeds, and grape skin, which contain a variety of biophenolic and flavonoid substances, have generated remarkable interest based on positive reports of their antioxidant properties and ability to serve as free radical scavengers [23]. Grapes have many types and a high content of polyphenolic compound. After being fermented into wine, the polyphenol content is increased, the composition is more stable, and the antioxidant capacity is greatly improved [24,25,26]. More than 50 polyphenolic compounds in red wine are the most widely used antioxidants. Grape skin contains more resveratrol than the grape meat and grape seed [27]. Resveratrol is a polyhydric phenolic compound. In addition to preventing cardiovascular and cerebrovascular diseases, it also has strong anticancer abilities [28]. Some clinical data have shown that procyanidin oligomers from grape seeds are 20 times more potent than vitamin C and 50 times more potent than vitamin E in antioxidants [29]. In addition to their antioxidant activity, grape skin polyphenols also inhibit some enzymes that catalyze the release of histamine, which is responsible for inflammation and allergies [30,31]. In vitro antioxidant studies have also confirmed that KFSE has a strong antioxidant capacity, whether for DPPH or ABTS. The fermentation solution components were separated by HPLC, and the results showed that the fermentation solution included EGCG, coumarin, neochlorogenic acid, rutin, resveratrol, chlorogenic acid, and rosmarinic acid. These ingredients have all been shown to have good antioxidant properties.

Oxidative stress is a negative effect produced by free radicals in the body. It refers to the imbalance of oxidation and antioxidation in the body, which tends to oxidize, leading to inflammatory infiltration of neutrophils, increased secretion of proteases, and the production of a large number of oxidized intermediates. Oxidative stress is considered an important factor leading to ageing and disease.

The superoxide anion reacts with hydrogen ions under the action of SOD to form hydrogen peroxide, which in turn reacts with hydrogen ions under the action of CAT and GSH-Px to finally form harmless substances such as water and oxygen [32]. LDH (lactate dehydrogenase) is a glycolytic enzyme widely distributed in the liver and kidney. An increase in LDH content indicates inflammation [33]. MDA is one of the end products of membrane lipid peroxide and can be used as one of the indicators of oxidative damage model. Depending on the severity of oxidative stress, NO can directly oxidize endogenous antioxidants, destroy non-enzymatic antioxidant defense systems, and inhibit the activity of antioxidant enzymes such as CAT and GSH-Px, leading to an increase in intracellular peroxide content and initiating cellular oxidative damage [34]. At the cellular level, KFSE can reduce H_2_O_2_-induced oxidative damage in human renal epithelial cells. In our study, we showed that the oxidative markers GSH, GSH-Px, CAT, SOD, and T-AOC were increased, and the content of LDH, MDA, and NO was decreased in cells. In addition, we demonstrated that KFSE was associated with increased oxidative damage in cells with increased expression of intracellular SOD, CAT, GSH-Px, and GSH genes and proteins. GSH as a strong reducing agent that can reduce part of the sulfhydryl form, thereby preventing the thiol from being contained [31]. Proteins and enzymes are protected from peroxide damage.

The genetic information on which organisms depend for survival and reproduction is stored in DNA, so maintaining the integrity of DNA molecules is critical to the survival of cells. The external environment and internal factors of the organism often lead to damage or changes in DNA molecules, which can affect the synthesis of RNA and protein. If the damage to DNA or the change in genetic information cannot be corrected, it may affect the cells. Cellular function or survival may affect germ cells and subsequent offspring.

The *p53* gene is closely related to the occurrence, development, and clinical treatment of tumors [35]. It is involved in the regulation of cell cycle, DNA repair, cell differentiation, apoptosis, and other anticancer biological functions [36,37,38]. The expression product of the *p21* gene is the most widely known kinase inhibitor. An active cell cycle inhibitory protein, *p21* inhibits the activity of *cyclin-D1*-*CDK4*, thereby arresting the cell cycle in G1 phase and inhibiting DNA replication [39]. Moreover, *p21* can decrease the expression of *PCNA* and arrest cells in S phase; *CDK4*, as a member of the kinase family, is a key regulator of cell transformation from G1 to S phase, and its content and activity level are closely related to the G1/S phase transformation rate of the cell cycle [40]. Elevated expression of *CDK4* promotes cell cycle progression and enhances cell proliferation, leading to tumorigenesis. This result was similarly confirmed in the flow cytometry results, and the cell cycle was arrested in the G1/S phase. In our study, the reduction in cell growth and cell cycle arrest by KFSE were accompanied by an increase in apoptotic genes and changes in cell cycle gene expression. In our cell-level studies, it was confirmed that KFSE promoted the expression of *p53*, *p21*, *cyclin-D1*, *CDK4*, *PCNA*, and other genes in cells when to the cells initiated apoptosis. *p53* induced upregulation of *p21* and other genes, which suggests that this change may play an important role in G1/S phase arrest caused by DNA damage.

At the protein level, KFSE induced up-regulation of Bax protein expression and decreased NF-κB protein expression. Bax is one of the most pro-apoptotic proteins in the human body. It can induce the release of Cytc and activate the caspase family of proteases, causing bubble formation, nuclear fragmentation and apoptosis, leading to DNA degradation [41,42,43]. After acting on HepG2 hepatoma cells, KFSE stimulates the death receptor pathway, and the death receptor is activated by binding to the corresponding ligand. After a series of downstream signal cascades, Caspase8 is activated, and the pro-apoptotic protein Bax is activated.

## 5. Conclusions

In conclusion, grape skin was fermented by *Lactobacillus plantarum* KFY02, and the variety of polyphenolic compounds increased. KFSE treatment induced cell cycle arrest via the *p53* and *p21* pathways involved in cell cycle progression and apoptosis, significantly inhibiting the growth of hepatoma cells in vitro. In addition, we demonstrated that the antioxidant capacity of KFSE manifested in vitro and had a beneficial effect on oxidative damage induced by H_2_O_2_ in renal epithelial cells. The *Lactobacillus plantarum* KFY02 fermented grape skin industry will have potential research value for the development of the future grape supplement industry and the development of functional foods for inhibiting hepatoma.

## Figures and Tables

**Table 1:** Flow phase gradient elution program.

t/min	A%	B%
0–20	97–95	3–5
20–35	95–85	5–15
35–50	85–70	15–30
50–65	70–70	30–30
65–75	70–0	30–0

**Table 2:** LDH, GSH, GSH-Px, CAT, MDA, SOD, T-AOC, and NO content in 293T cells.

Group	LDH (U/10^4^)	GSH (μg/10^4^)	GSH-Px (U/mg prot)	CAT (U/10^4^)	MDA (nmol/10^4^)	SOD (U/10^4^)	T-AOC (U/10^4^)	NO (μmol/10^4^)
**Normal**	5.44 ± 0.16 ^a^	3.13 ± 0.14 ^c^	2.50 ± 0.20 ^c^	2.77 ± 0.21 ^c^	1.42 ± 0.04 ^a^	18.12 ± 0.61 ^d^	18.32 ± 0.79 ^d^	4.20 ± 0.39 ^a^
**Control**	8.90 ± 0.33 ^c^	1.89 ± 0.07 ^a^	0.91 ± 0.11 ^a^	1.43 ± 0.10 ^a^	2.72 ± 0.17 ^c^	10.46 ± 0.57 ^a^	11.42 ± 0.16 ^a^	8.43 ± 0.49 ^c^
**KFSE**	6.56 ± 0.64 ^b^	2.72 ± 0.07 ^b^	1.80 ± 0.07 ^b^	2.06 ± 0.09 ^b^	1.68 ± 0.06 ^b^	16.99 ± 0.19 ^c^	15.92 ± 0.25 ^c^	5.62 ± 0.12 ^b^
**BFSE**	7.40 ± 0.45 ^b^	2.76 ± 0.27 ^b^	1.62 ± 0.08 ^b^	1.95 ± 0.11 ^b^	1.75 ± 0.09 ^b^	16.63 ± 0.32 ^c^	14.62 ± 1.22 ^b^	4.38 ± 0.66 ^a^
**WS**	7.41 ± 0.40 ^b^	2.67 ± 0.24 ^b^	1.72 ± 0.03 ^b^	1.88 ± 0.05 ^b^	1.79 ± 0.08 ^b^	15.73 ± 0.47 ^b^	13.40 ± 0.31 ^b^	5.29 ± 0.25 ^b^

^a–d^ Mean values with different letters in the same column differ significantly (*p* < 0.05) by Duncan′s multiple range test. Values presented are the means ± standard deviation (*N* = 3/group). KFSE: The fermentation solution fermented by LP-KFY02; BFSE: The fermentation solution fermented by *Lactobacillus delbrueckii* subsp. *bulgaricus*; and WS: The solution extracted by ethanol.

**Table 3:** The mRNA expression of *SOD*, *GSH*, *CAT* and *GSH-Px* in 293T cells.

Group	*CAT*	*SOD*	*GSH*	*GSH-Px*
**Normal**	1.01 ± 0.18 ^c^	1.00 ± 0.09 ^d^	1.00 ± 0.09 ^c^	1.01 ± 0.18 ^c^
**Control**	0.39 ± 0.08 ^a^	0.12 ± 0.01 ^a^	0.32 ± 0.03 ^a^	0.35 ± 0.04 ^a^
**KFSE**	1.11 ± 0.08 ^c^	0.57 ± 0.09 ^c^	1.07 ± 0.13 ^c^	1.22 ± 0.08 ^c^
**BFSE**	0.66 ± 0.13 ^b^	0.30 ± 0.03 ^b^	0.97 ± 0.08 ^c^	0.72 ± 0.14 ^b^
**WS**	0.66 ± 0.04 ^b^	0.28 ± 0.08 ^b^	0.81 ± 0.06 ^b^	0.54 ± 0.16 ^a,b^

^a–d^ Mean values with different letters in the same column differ significantly (*p* < 0.05) by Duncan’s multiple range test. Values presented are the means ± standard deviation (*N* = 3/group). KFSE: The fermentation solution fermented by LP-KFY02; BFSE: The fermentation solution fermented by *Lactobacillus delbrueckii* subsp. *bulgaricus*; and WS: The solution extracted by ethanol.

**Table 4:** The mRNA expression of *Bcl-2*, *cox-2*, *Caspase-3*, *Caspase-7*, and *Caspase-8* in HepG2 cells.

Group	*Bcl-2*	*cox-2*	*Caspase-3*	*Caspase-7*	*Caspase-8*
**Normal**	1.01 ± 0.16 ^c^	1.01 ± 0.13 ^b^	1.01 ± 0.13 ^a^	1.00 ± 0.05 ^a^	1.13 ± 0.61 ^a^
**KFSE**	0.60 ± 0.08 ^a^ ↓	0.72 ± 0.10 ^a^ ↓	2.25 ± 0.56 ^b^ ↑	3.39 ± 0.39 ^c^ ↑	3.52 ± 1.07 ^b^ ↑
**BFSE**	0.92 ± 0.11b ^c^ ↓	0.76 ± 0.03 ^a^ ↓	1.90 ± 0.37 ^b^ ↑	1.14 ± 0.18 ^a^ ↑	2.57 ± 1.87 ^a,b^ ↑
**WS**	0.81 ± 0.07 ^b^ ↓	0.76 ± 0.04 ^a^ ↓	1.77 ± 0.08 ^b^ ↑	2.57 ± 0.88 ^b^ ↑	1.43 ± 0.13 ^a^ ↑

^a–d^ Mean values with different letters in the same column differ significantly (*p* < 0.05) by Duncan’s multiple range test. Values presented are the means ± standard deviation (*N* = 3/group). KFSE: The fermentation solution fermented by LP-KFY02; BFSE: The fermentation solution fermented by *Lactobacillus delbrueckii* subsp. *bulgaricus*; and WS: The solution extracted by ethanol.

**Table 5:** The mRNA expression of *TGF-β*, *Caspase-9*, *C-Myc*, *CyclinD1*, and *CDK4* in HepG2 cells.

Group	*TGF-β*	*Caspase-9*	*C-myc*	*cyclin-D1*	*CDK4*
**Normal**	1.14 ± 0.24 ^a^	1.00 ± 0.10 ^a^	1.00 ± 0.10 ^a^	1.00 ± 0.11 ^c^	1.00 ± 0.10 ^d^
**KFSE**	3.18 ± 0.17 ^c^ ↑	2.52 ± 0.57 ^d^ ↑	1.19 ± 0.13 ^a^ ↑	0.22 ± 0.05 ^a^ ↓	0.31 ± 0.04 ^a^ ↓
**BFSE**	5.41 ± 0.87 ^c^ ↑	1.39 ± 0.39 ^b^ ↑	1.11 ± 0.24 ^a^ ↑	0.36 ± 0.03 ^b^ ↓	0.60 ± 0.06 ^c^ ↓
**WS**	3.71 ± 1.07 ^b^ ↑	1.92 ± 0.18 ^c^ ↑	1.17 ± 0.17 ^a^ ↑	0.19 ± 0.12 ^a^ ↓	0.49 ± 0.04 ^b^ ↓

^a–d^ Mean values with different letters in the same column differ significantly (*p* < 0.05) by Duncan’s multiple range test. Values presented are the means ± standard deviation (*N* = 3/group). KFSE: The fermentation solution fermented by LP-KFY02; BFSE: The fermentation solution fermented by *Lactobacillus delbrueckii* subsp. *bulgaricus*; and WS: The solution extracted by ethanol.

**Table 6:** The mRNA expression of *NF-κB*, *p21*, *p53*, *PCNA*, and *pRB1* in HepG2 cells.

Group	*NF-κB*	*p21*	*p53*	*PCNA*	*qRB1*
**Normal**	0.91 ± 0.24 ^b^	0.90 ± 0.26 ^a^	0.89 ± 0.34 ^a^	1.00 ± 0.06 ^b^	1.00 ± 0.05 ^c^
**KFSE**	0.58 ± 0.08 ^a^ ↓	4.48 ± 0.30 ^c^ ↑	2.27 ± 0.25 ^b^ ↑	0.18 ± 0.03 ^a^ ↓	0.15 ± 0.05 ^a^ ↓
**BFSE**	0.63 ± 0.09 ^a^ ↓	1.06 ± 0.12 ^a^ ↑	1.18 ± 0.30 ^a^ ↑	0.19 ± 0.01 ^a^ ↓	0.13 ± 0.02 ^a^ ↓
**WS**	0.57 ± 0.11 ^a^ ↓	2.66 ± 0.06 ^b^ ↑	1.97 ± 0.18 ^b^ ↑	0.21 ± 0.01 ^a^ ↓	0.26 ± 0.05 ^b^ ↓

^a–d^ Mean values with different letters in the same column differ significantly (*p* < 0.05) by Duncan’s multiple range test. Values presented are the means ± standard deviation (*N* = 3/group). KFSE: The fermentation solution fermented by LP-KFY02; BFSE: The fermentation solution fermented by *Lactobacillus delbrueckii* subsp. *bulgaricus*; and WS: The solution extracted by ethanol.

## Appendix A

**Table A1:** Sequences of primers used in this study.

Gene	Primer Sequence	X (Annealing Temperature (°C))
***GAPDH***	F: 5′-TCAAGAAGGTGGTGAAGCAGG-3′ R: 5′-AGCGTCAAAGGTGGAGGAGTG-3′	
***CAT***	F: 5′-TGGAGCTGGTAACCCAGTAGG-3′ R: 5′-CCTTTGCCTTGGAGTATTTGGTA-3′	57.0
***SOD1***	F: 5′-GGTGGGCCAAAGGATGAAGAG-3′ R: 5′-CCACAAGCCAAACGACTTCC-3′	57.0
***GSH***	F: 5′-GGGAGCCTCTTGCAGGATAAA-3′ R: 5′-GAATGGGGCATAGCTCACCAC-3′	57.0
***GSH-Px***	F: 5′-CAGTCGGTGTATGCCTTCTCG-3′ R: 5′-GAGGGACGCCACATTCTCG-3′	57.0
***p53***	F: 5′-CTTTGAGGTGCGTGTTTGTGC-3′ R: 5′-GGTTTCTTCTTTGGCTGGGGA-3′	51.6
***CDK4***	F: 5′-ATGGCTACCTCTCGATATGAGC-3′ R: 5′-CATTGGGGACTCTCACACTCT-3′	56.6
***Cyclin-D1***	F: 5′-GCTGCGAAGTGGAAACCATC-3′ R: 5′-CCTCCTTCTGCACACATTTGAA-3′	60.3
***p21***	F: 5′-TGTCCGTCAGAACCCATGC-3′ R: 5′-AAAGTCGAAGTTCCATCGCTC-3′	54.9
***pRb1***	F: 5′-CTCTCGTCAGGCTTGAGTTTG-3′ R: 5′GACATCTCATCTAGGTCAACTGC-3′	66.0
***C-myc***	F: 5′-TGGAACGTCAGAGGAGAAACGA-3′ R: 5′-CTTGAACGGACAGGATGTAGGC-3′	50.0
***Caspase-3***	F: 5′-CATGGAAGCGAATCAATGGACT-3′ R: 5′-CTGTACCAGACCGAGATGTCA-3′	50.7
***Caspase-8***	F: 5′-ATTTTGAGATCAAGCCCCACG-3′ R: 5′-GGATACAGCAGATGAAGCAGTCC-3′	66.0
***Caspase-9***	F: 5′-CTCAGACCAGAGATTCGCAAAC-3′ R: 5′-GCATTTCCCCTCAAACTCTCAA-3′	50.0
***Caspase-7***	F: 5′-CGGTCCTCGTTTGTACCGTC-3′ R: 5′-CGCCCATACCTGTCACTTTATCA-3′	64.4
***cox-2***	F: 5′-CTGGCGCTCAGCCATACAG-3′ R: 5′-CGCACTTATACTGGTCAAATCCC-3′	65.0
***Bcl-2***	F: 5′-ATGTGTGTGGAGAGCGTCAACC-3′ R: 5′-CAGAGACAGCCAGGAGAAATCAA-3′	65.0
***NF-κB***	F: 5′-GAAGCACGAATGACAGAGGC-3′ R: 5′-GCTTGGCGGATTAGCTCTTTT-3′	50.9
***PCNA***	F: 5′-CCAGGGCTCCATCCTCAAGAA-3′ R: 5′-GACGTGGGACGAGTCCATGCT-3′	62.0
***TGF-β***	F: 5′-CAGCGTGCCTAAACTTTATCAGC-3′ R: 5′-TCAGGAGGATGTTTCACATGGA-3′	58.2
